# The Intraoperative Administration of Dexmedetomidine Alleviates Postoperative Inflammatory Response in Patients Undergoing Laparoscopy-Assisted Gastrectomy: A Double-Blind Randomized Controlled Trial

**DOI:** 10.3390/biomedicines11123253

**Published:** 2023-12-08

**Authors:** Jiae Moon, Duk-Hee Chun, Hee Jung Kong, Hye Sun Lee, Soyoung Jeon, Jooeun Park, Na Young Kim, Hyoung-Il Kim

**Affiliations:** 1 Department of Anesthesiology and Pain Medicine, Anesthesia and Pain Research Institute, Yonsei University College of Medicine, Seoul 03722, Republic of Korea; answldo@yuhs.ac (J.M.); sarajo8768@yuhs.ac (H.J.K.); rlo2@yuhs.ac (J.P.); 2 Department of Anesthesiology and Pain Medicine, CHA Bundang Medical Center, CHA University, Seongnam 13496, Republic of Korea; 3 Department of Research Affairs, Biostatistics Collaboration Unit, Yonsei University College of Medicine, Seoul 03722, Republic of Korea; hslee1@yuhs.ac (H.S.L.); jsy0331@yuhs.ac (S.J.); 4 Department of Surgery, Yonsei University College of Medicine, Seoul 03722, Republic of Korea

**Keywords:** laparoscopy-assisted gastrectomy, dexmedetomidine, inflammatory response, interleukin-6

## Abstract

Surgical stress can compromise the immune system of patients with cancer, affecting susceptibility to perioperative infections, tumor progression, treatment responses, and postoperative recovery. Perioperatively reducing inflammatory responses could improve outcomes. We determined the impact of intraoperative dexmedetomidine administration on the inflammatory response and postoperative recovery in patients undergoing elective laparoscopy-assisted gastrectomy. These patients were randomly assigned to the dexmedetomidine or control group (*n* = 42 each). The primary endpoint was the C-reactive protein (CRP) level on postoperative day 1. The secondary endpoints included the perioperative interleukin (IL)-6 levels, postoperative numerical rating scale (NRS) scores, and rescue analgesic doses. There were no significant between-group differences in terms of CRP levels. The IL-6 levels at the end of the surgery, NRS scores in the post-anesthesia care unit, and rescue pethidine requirements within the first hour postoperatively were significantly lower in the dexmedetomidine group than in the control group. The bolus deliveries-to-attempts ratio (via patient-controlled analgesia) at 2 h differed significantly between the two groups. However, IL-6 reduction was confined to a single timepoint, and the postoperative analgesic effects lasted for the first 2 h postoperatively. Low-dose dexmedetomidine infusion (0.4 µg kg^−1^ h^−1^) during laparoscopy-assisted gastrectomy exerts minimal anti-inflammatory effects.

## 1. Introduction

The physiological inflammatory response to surgical stress in patients with cancer induces dysfunction of their immune system. This may increase their susceptibility to perioperative infections, which reportedly have a significant impact on tumor development, progression, and response to therapy. The inflammatory response can also delay postoperative recovery [1,2,3,4,5,6]. Certain systemic inflammatory markers, such as C-reactive protein (CRP), tumor necrosis factor alpha (TNF-α), interleukin-6 (IL-6), neutrophil-to-lymphocyte ratio (NLR), and platelet-to-lymphocyte ratio, are known to play a prognostic role in various types of cancer following surgery [1,2,3]. Recent studies have shown that perioperative strategies can significantly influence the postoperative outcomes in patients with cancer [4,5,6]. One such strategy is to reduce the production of inflammatory factors, which would then reduce immune dysfunction and lead to favorable outcomes in these patients [6,7].

Dexmedetomidine, a highly selective α_2_-adrenergic agonist with sedative and analgesic properties, exerts immunomodulatory effects, reduces stress responses, and demonstrates anti-inflammatory activity [8,9,10]. Its administration reportedly reduces the IL-6 and TNF-α levels in animals with severe inflammation [9,10], and the IL-6, TNF-α, and CRP levels in humans undergoing surgery [8]. Thus, the perioperative administration of dexmedetomidine is expected to yield beneficial outcomes in patients undergoing surgery.

This double-blind, randomized, controlled trial aimed to determine whether the intraoperative administration of dexmedetomidine exerted anti-inflammatory effects in patients undergoing laparoscopy-assisted gastrectomy, with a focus on the postoperative inflammatory markers CRP and IL-6. Furthermore, the effects of intraoperative dexmedetomidine administration on postoperative recovery, and on postoperative analgesia in particular, were also evaluated.

## 2. Materials and Methods

### 2.1. Patients

This double-blind, randomized clinical trial was approved by the Institutional Review Board (IRB) and the Hospital Research Ethics Committee of Severance Hospital, Yonsei University Health System (Seoul, Republic of Korea; IRB no.: 4-2019-0055). Prior to patient enrolment, the trial was registered at Clinical trials (NCT03960775). Written informed consent was obtained from all participants before enrolment.

This study enrolled adult patients aged 20–70 years who were scheduled to undergo an elective laparoscopy-assisted gastrectomy under general anesthesia at the Yonsei University Severance Hospital between April and November 2019. The exclusion criteria were as follows: body weight ≥90 kg or body mass index ≥32 kg m^−2^; history of gastric surgery or any other complicated surgery involving the resection of other organs; presence of unstable angina, congestive heart failure, arrhythmia, uncontrolled hypertension, or bradycardia; history of cerebral hemorrhage or cerebral ischemia; use of antiarrhythmic agents; presence of an uncontrolled psychological disorder; inability to communicate.

### 2.2. Study Design

Using a randomization table, patients were randomly assigned to the control group (*n* = 42) or the dexmedetomidine group (*n* = 42). The randomization code was generated using a randomization program (www.random.org). In the dexmedetomidine group, participants received a continuous infusion of dexmedetomidine (100 µg mL^−1^ in a 2 mL-vial; HospiraWorldwide, Seoul, Republic of Korea) at a rate of 0.4 μg kg^−1^ h^−1^ using an infusion pump (Terufusion TE-311, Terumo, Tokyo, Japan). The infusion was initiated immediately after the induction of anesthesia and was continued until the closure of the peritoneum. In the control group, participants received a continuous infusion of 0.9% normal saline at a rate of 0.4 μg kg^−1^ h^−1^ using the same protocol. The study medications were prepared by a nurse, who was not involved in this study, after referring to a randomization table; both dexmedetomidine and normal saline appeared transparent and were indistinguishable. The surgeons, patients, recovery nurses, and attending anesthesiologists, as well as the investigator, were blinded to the group allocation.

### 2.3. Anesthetic Management Procedures

Anesthetic management was identical in both groups. In brief, 1–1.5 mg kg^−1^ of propofol was administered and 0.2 μg kg^−1^ min^−1^ of remifentanil was infused continuously for the induction of anesthesia. Following the loss of consciousness, 0.6 mg kg^−1^ of rocuronium was administered for neuromuscular blockade. The patients were intubated after muscle relaxation. Anesthesia was maintained using desflurane; furthermore, the target Bispectral Index™ (Medtronic Ltd., Minneapolis, MN, USA) was maintained between 40 and 60 while ensuring the continuous administration of remifentanil and rocuronium. The remifentanil infusion rate was adjusted to maintain an adequate blood pressure and heart rate. Mechanical ventilation was set in the volume control mode, with a target tidal volume of 8 mL kg^−1^ and target end-tidal CO_2_ of 35–45 cmH_2_O. Patients were extubated once they recovered adequate respiration and consciousness; they were then transferred to the post-anesthesia care unit (PACU).

### 2.4. Postoperative Pain Management

Before the operation ended, we administered 1 μg kg^−1^ of fentanyl (Hana Pharm, Seoul, Korea) for immediate postoperative analgesia and 0.3 mg of ramosetron (Nasea^®^, Astellas Pharma Korea, Seoul, Republic of Korea) for prophylaxis against postoperative nausea and vomiting. For postoperative pain control, we opted for intravenous patient-controlled analgesia (PCA; Accumate 1200; WooYoung Medical, Seoul, Republic of Korea) with fentanyl (1500 µg) and ramosetron (0.3 mg) in a 150 mL saline solution; this solution was administered under the following conditions: basal rate, 1 mL h^−1^; bolus, 1 mL; and lockout time interval, 3 min. Patients who experienced sustained pain (numerical rating scale [NRS] score ≥4) were administered pethidine (25 mg; Pethidine inj, Jeil Pharm, Seoul, Republic of Korea) as an additional analgesic until 48 h postoperatively.

### 2.5. Outcomes and Data Collection

The primary endpoint was the effect of dexmedetomidine on the CRP levels on postoperative day (POD) 1 in patients who underwent laparoscopy-assisted gastrectomy. The secondary endpoints were the perioperative IL-6 levels, white blood cell (WBC) count, and NLR; the postoperative NRS score; and the rescue analgesic dose administered until 48 h postoperatively.

The CRP level, WBC count, and NLR were measured at the following timepoints: preoperative outpatient visit (pre-OP), POD 0, POD 1, POD 2, POD 3, POD 5, 1 month postoperatively, and 3 months postoperatively. Blood sampling for IL-6 estimation was performed at the following three timepoints: after the induction of anesthesia (pre-OP), 90 min after the start of pneumoperitoneum (Pneumo 90 min), and at the end of the surgery (OP end). The collected blood samples were immediately centrifuged at 5000 rpm for 5 min at 4 °C to separate the plasma; the collected plasma was then stored at −80 °C until analysis. The plasma IL-6 levels were measured using a specific immunoassay kit (R&D, Cat. No. D6050, Irving, TX, USA), and all measurements were performed in duplicate.

Data on the following patient and surgical characteristics were collected: age, sex, body mass index, American Society of Anesthesiologists physical status, comorbidities (such as hypertension or diabetes mellitus), type of operation, type of reconstruction and anastomosis, extent of lymph node dissection, and tumor-node-metastasis stage. Data on the following intraoperative variables were collected: duration of anesthesia and surgery, pneumoperitoneum time, intraoperative fluid intake, blood loss, urine output, dose of remifentanil administered, dose of ephedrine administered, and total amount of phenylephrine infused. Data on the following postoperative variables were collected: length of hospital stay, final pathology, surgical complications, and PCA-related complications.

The postoperative NRS score and rescue pethidine dose were determined in the PACU and at 1, 2, 4, 8, 12, 24, and 48 h postoperatively. The total volume of fentanyl delivered, number of times the patient attempted to administer a bolus (i.e., attempts), and the actual number of boluses administered (i.e., deliveries) were evaluated using data recorded in the PCA device.

### 2.6. Statistical Analysis

In a previous study on patients with gastric cancer who underwent radical resection with or without an intraoperative dexmedetomidine infusion, researchers found that the CRP concentration in the dexmedetomidine group on POD 1 was 20.62 ± 3.56 (mg L^−1^) [11]. Furthermore, in a preliminary retrospective study (IRB no.: 4-2018-0257), the CRP level in the control group on POD 1 was found to be 35.49 ± 22.38 (mg L^−1^). Accordingly, the mean difference in the CRP level on POD 1 between the control and dexmedetomidine groups was 14.87 (mg L^−1^). Therefore, 37 participants were required in each group to provide a power of 0.8 and an α error of 0.05; thus, after considering a dropout rate of 10%, we planned to recruit 84 patients (42 per group)

Continuous data were tested for normality using the Shapiro–Wilk test. Parametric data are expressed as mean ± standard deviation (SD), whereas non-parametric data are expressed as median (interquartile range); categorical data are expressed as numbers and percentages. Parametric data were compared using an independent *t*-test, whereas nonparametric data were compared using the Mann–Whitney *U* test. Categorical data were compared using the χ^2^ test or the Fisher’s exact test. Repeatedly measured data, such as the CRP level, WBC count, hematocrit, cytokine levels, and postoperative pain scores, were analyzed using a linear mixed model. Statistical significance was set at *p* < 0.05. All data were analyzed using SPSS version 23.0 and SAS version 9.2 (SAS Inc., Cary, NC, USA).

## 3. Results

### 3.1. Patients

**Figure 1:**
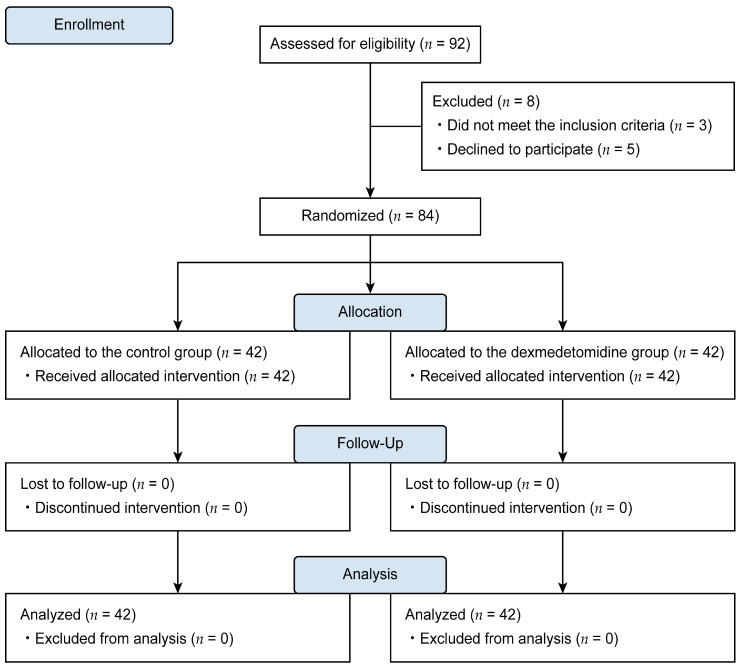
CONSORT flow diagram.

A total of 92 patients were assessed for eligibility; among these, 84 met the inclusion criteria and agreed to participate in the study. Data from these patients (treatment group, *n* = 42; control group, *n* = 42) were included in the final analysis (Figure 1).

### 3.2. Demographic and Perioperative Characteristics

Data on the patient characteristics and surgical variables are presented in Table 1; these did not differ significantly between the two groups.

Data on the intraoperative variables are presented in Table 2. The intraoperative doses of remifentanil and ephedrine were significantly lower and higher, respectively, in the dexmedetomidine group than in the control group. No significant differences in the other variables were noted between the two groups.

The postoperative data (including the length of hospital stay) also did not differ between the two groups (Table 3).

### 3.3. Inflammatory Indices: IL-6, CRP, WBC, and NLR

**Figure 2:**
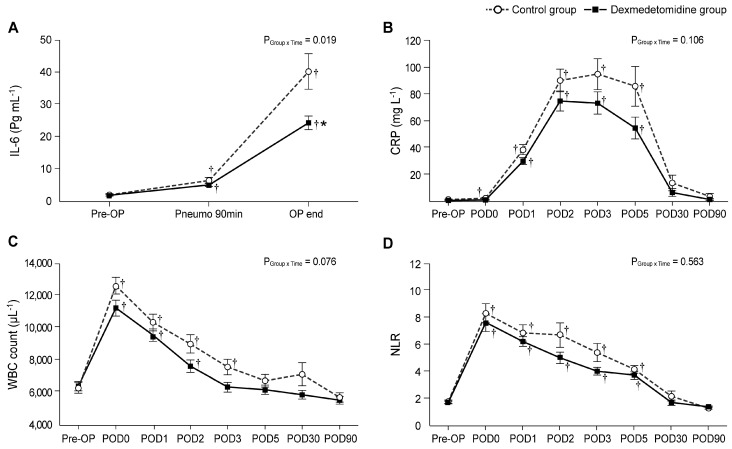
Perioperative changes in the inflammatory indices: (**A**) IL-6, (**B**) CRP, (**C**) WBC, and (**D**) NLR. Pre-OP, pre-operation; Pneumo 90 min, 90 min after the start of pneumoperitoneum; OP end, at the end of the surgery; POD, postoperative day; IL-6, interleukin-6; CRP, C-reactive protein; WBC, white blood cell; NLR, neutrophil-to-lymphocyte ratio. Values are shown as mean ± standard deviation. † Bonferroni-corrected *p* < 0.05 versus pre-OP in each group. * Bonferroni-corrected *p* < 0.05 versus the control group.

Table 4 and Figure 2 shows the perioperative changes in the IL-6 level, CRP level, WBC count, and NLR. As seen in Figure 2A, even though the baseline values of IL-6 level were similar between the two groups, significant intergroup differences were observed over time (P_Group × Time_ = 0.019). The IL-6 level in the dexmedetomidine group was significantly lower than in the control group in the OP end (22.0 ± 12.5 ng/mL vs. 36.2 ± 32.1 ng/mL; Bonferroni corrected *p* = 0.018). When compared with its baseline value, the IL-6 level was significantly increased throughout all the timepoints (Pneumo 90 min and OP end) in both groups.

Significant differences in CRP levels were not found between the two groups using linear mixed-model analysis (P_Group × Time_ = 0.106). When compared with the baseline values, in the control group, the CRP levels on PODs 0, 1, 2, 3, and 5 were significantly higher than those in the pre-OP (baseline; Figure 2B). Conversely, in the dexmedetomidine group, the CRP levels did not differ significantly between pre-OP and POD 0; however, they were significantly increased on PODs 1, 2, 3, and 5 compared to the pre-OP value. The CRP level was lower in the dexmedetomidine group than in the control group; however, this difference was not significant.

The WBC counts at POD 0 were significantly higher than those at pre-OP in both the control and the dexmedetomidine group; furthermore, they continued to increase until POD 2 and POD 3 in the dexmedetomidine and control groups, respectively (Figure 2C). However, no significant intergroup difference was found (P_Group × Time_ = 0.076).

As seen in Figure 2D, the NLRs in both groups were significantly higher from POD 0 to POD 5 compared to the values of the preoperative baseline, but there was no significant difference between the two groups.

### 3.4. Postoperative Pain Scores and Analgesic Requirements

**Figure 3:**
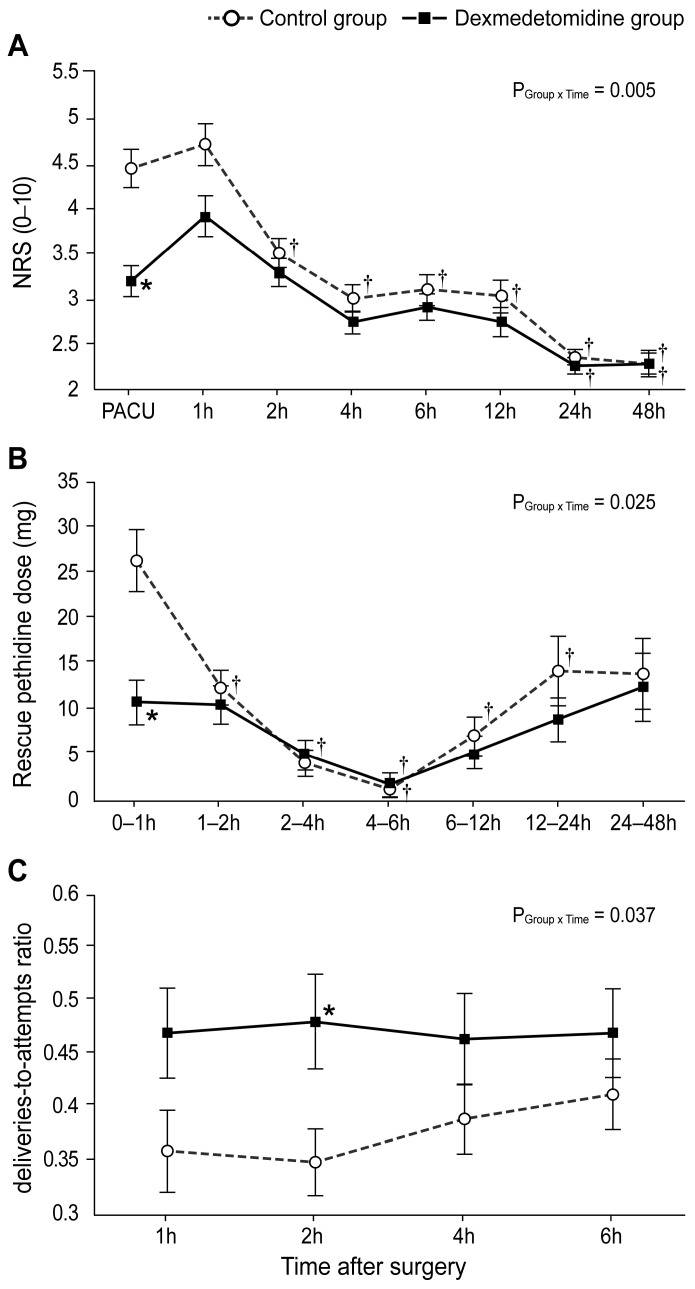
Postoperative pain scores and analgesic requirement: (**A**) Numerical rating scale score for postoperative pain intensity during 48 h after the operation. (**B**) Dose of rescue pethidine during 48 h after the operation. (**C**) The ratio of bolus deliveries-to-attempts via patient-controlled analgesia. Values are shown as mean ± standard deviation. † Bonferroni-corrected *p* < 0.05 versus pre-OP in each group. * Bonferroni-corrected *p* < 0.05 versus the control group.

As seen in Figure 3A, the NRS scores (evaluated in the PACU) were significantly lower in the dexmedetomidine group than in the control group (Bonferroni-corrected *p* < 0.005). Furthermore, the rescue pethidine requirement within the first hour was significantly lower in the dexmedetomidine group than in the control group (Bonferroni-corrected *p* = 0.003; Figure 3B). The ratio of bolus deliveries-to-attempts via PCA at 2 h postoperatively differed significantly between the two groups (Bonferroni-corrected *p* = 0.047, Figure 3C).

## 4. Discussion

This prospective, double-blind, randomized controlled trial determined the effects of continuous intraoperative dexmedetomidine administration on the inflammatory response and postoperative recovery in patients undergoing laparoscopy-assisted gastrectomy. Compared to controls, patients who received an infusion of a clinically low dose of dexmedetomidine (0.4 μg kg^−1^ h^−1^) during anesthesia did not exhibit significantly different CRP levels on POD 1.

The inflammatory response elicited by surgery induces immune cell changes that lead to cell degeneration, necrosis, and dysfunction in various organs [12]. This response, initiated by macrophages and monocytes at the site of the injury, involves a complex biochemical cascade that includes the release of cytokines such as TNF-α, IL-1β, and IL-6 [13]. Among these inflammatory markers, IL-6 serves as a primary proinflammatory cytokine that is produced within 2–4 h after a tissue injury, making it an early and valuable independent marker [14]. CRP is primarily produced by the liver in response to IL-6 and shows good correlation with the IL-6 levels. It is secreted within 4–12 h after stimulation and reaches a peak 24–72 h later [15]. The physiological response to surgical stress not only induces immune system dysfunction and predisposes patients to perioperative infections, but also delays postoperative recovery [16,17,18,19,20]. Therefore, reducing inflammation in patients undergoing surgery might improve their postoperative clinical outcomes and prognoses.

The anti-inflammatory effects of dexmedetomidine are well-established in laboratory and clinical settings [21,22,23,24,25]. Previous studies have shown that dexmedetomidine decreases the release of stress response biomarkers [21] and suppresses sympathetic nerve hyperactivity [26,27]. Furthermore, dexmedetomidine infusion was shown to suppress the serum IL-6, TNF-α, and CRP production in patients undergoing surgery and attenuate their surgical stress response [7,11,21,22,23]. However, while we observed a significant difference in the IL-6 levels at the end of the surgery, the CRP levels on POD 1 did not differ significantly between the dexmedetomidine and control groups; this is inconsistent with the abovementioned findings. In our study, the IL-6 values at the end of the operation differed significantly between the dexmedetomidine and control groups; however, the CRP levels on POD 1 did not. This is inconsistent with the abovementioned findings. However, similar to us, Lee et al. also found that dexmedetomidine (administered at a rate of 0.4 mcg kg^−1^ h^−1^) did not alleviate the inflammatory responses characterized by CRP and cytokines (such as TNF-α, IL-6, and IL-10) in patients undergoing laparoscopic hysterectomy [28]. Additionally, Bekker et al. reported that the infusion of 0.5 mcg kg^−1^ h^−1^ of dexmedetomidine in patients undergoing multilevel spinal fusion did not reduce the elevation in the CRP level on POD 1, IL-6 level in the PACU and POD 1 (as compared to in the control group) [29]. However, they analyzed various other inflammatory markers, including cortisol, IL-1, IL-6, IL-8, IL-10, and TNF-α, and found that dexmedetomidine infusion led to a reduction in the plasma levels of cortisol and IL-10 (as compared to those in the control group). The inconclusive results on the anti-inflammatory effects of dexmedetomidine in these studies may be attributed to differences in the analyzed parameters, such as the surgical characteristics, total amount of dexmedetomidine administered, presence or absence of an initial loading dose, infusion rate of the maintenance dose, and duration of dexmedetomidine infusion. Furthermore, the degree of the anti-inflammatory effect exerted by dexmedetomidine varied among studies, especially in cases of excessive inflammatory reactions [30,31,32,33].

The degree of inflammation varies across clinical studies depending on the extent of the surgery, type of surgery, and the surgical technique used [20,30,34]. Alterations in the immune function and stress responses are proportional to the extent of the injury, and less invasive surgical techniques cause less surgical trauma and further alleviate inflammatory responses [35,36]. Short-term postoperative immune and inflammatory functions are reportedly better after a laparoscopic surgery than after the conventional open approach [20,34,37]. Furthermore, the IL-6 levels have been reported to be significantly lower with laparoscopic colectomy than with the open technique [20,38]. Previous studies on radical gastrectomy revealed a significant decrease in the IL-6 levels in patients who received dexmedetomidine at the end of the surgery in patients who received both the loading and maintenance [11,23]. In the present study, the less invasive laparoscopic approach may have attenuated inflammation; consequently, a low maintenance dose of dexmedetomidine was sufficient for suppressing IL-6 elevation at the end of the surgery.

Xie et al. conducted a study on patients with lung cancer undergoing a thoracoscopic radical resection with or without dexmedetomidine infusion; they observed that, among all parameters, cytokines (including IL-6) exhibited the greatest difference between the groups at 1 h after one-lung ventilation. Furthermore, the concentration of cytokines decreased at this timepoint. Therefore, we expected that IL-6 levels would decrease 90 min after pneumoperitoneum formation in our study; however, our findings indicated that the IL-6 levels increased until after the surgery. Unlike in previous studies, the initial loading dose was not considered in the present study [39]. Studies have suggested the need for an initial loading dose to promptly attenuate inflammation; the absence of an initial loading dose lengthens the time required to reach the effective plasma concentration of dexmedetomidine that suppresses the inflammatory response. However, unwanted complications (such as hypertension, hypotension, and bradycardia) can be avoided if the initial loading dose is not administered. Although the administered dose of ephedrine was significantly higher in the dexmedetomidine group than in the control group, no cases of severe bradycardia or hypotension were observed in the dexmedetomidine group. The effectiveness of a maintenance dose of dexmedetomidine against IL-6 could have been further demonstrated had we sought to assess the intergroup differences in the IL-6 levels after the surgery. Other inflammatory indices, such as the CRP level, WBC count, and NLR, did not differ significantly between the dexmedetomidine and control groups at all time points; a dose–response study would be necessary to determine the effective maintenance dose required to attenuate inflammatory factors during and after the surgery.

Dexmedetomidine reduces the release of stress-response biomarkers and is a useful adjuvant to anesthetics and opioids [7,22,40,41,42]. It further reduces anesthetic requirements, improves analgesia, reduces opioid use, and delays postoperative analgesic use [43,44,45,46]. In the present study, the NRS score in the PACU differed significantly between the dexmedetomidine and control groups. Furthermore, a significantly greater reduction in the rescue analgesic requests was noted within the first hour postoperatively in the dexmedetomidine group than in the control group. However, these differences were transient and were only observed during the immediate postoperative period. Furthermore, even though the deliveries-to-attempts ratio at 2 h postoperatively differed significantly between the two groups, the NRS score and rescue pethidine dose did not. These findings suggest that the analgesia provided by dexmedetomidine did not significantly improve the overall postoperative state. Dexmedetomidine has a half-life of approximately 2 h and remains pharmacologically effective after infusion is terminated at the end of the surgery [47]. However, in this study, the analgesic effect of dexmedetomidine began to dissipate at 2 h after discontinuation; this was shorter than the duration observed in other studies [40,48]. This difference may be attributed to the low clinical dose of dexmedetomidine administered in this study. Nevertheless, our findings indicate that a low dose of dexmedetomidine infused intraoperatively without an initial loading dose provided prolonged postoperative analgesia in patients undergoing laparoscopy-assisted gastrectomy.

This study had some limitations. First, this was a single-center study with a small sample size. The patients were observed only during a short perioperative period. Because inflammatory factors were assessed using laboratory tests only at designated timepoints, values may have been missed at certain other timepoints. The effect of dexmedetomidine on IL-6 could have been assessed more clearly had the IL-6 level been measured 2 h after termination of infusion or on POD 1. Additionally, because variations in the types and duration of stressful surgical manipulation can affect the inflammatory response, it is crucial to consider these factors and interpret the results with caution. Second, the effect of only one maintenance dose of dexmedetomidine was evaluated in this study. The effects of dexmedetomidine administered at various concentrations and infusion rates, with or without initial loading, will be determined in further dose–response studies. Third, the postoperative IL-6 levels were lower in the dexmedetomidine group than in the control group; however, no intergroup differences in the clinical outcomes, such as surgical complications or length of hospital stay, were noted. In addition, we only assessed the adverse complications over a short-term period. Additional studies should be conducted to determine whether low-dose dexmedetomidine (rather than laboratory findings alone) has a positive effect on the long-term clinical outcomes after surgery.

## 5. Conclusions

The intraoperative administration of low-dose dexmedetomidine (0.4 µg kg^−1^ h^−1^) in patients undergoing laparoscopy-assisted gastrectomy did not achieve a significant reduction in the postoperative CRP level (relative to that in the control group). Although the IL-6 elevation was reduced at the end of the surgery and the immediate postoperative recovery indices were improved, the IL-6 reduction was limited to a single timepoint and the postoperative analgesic effects were confined to the first 2 h postoperatively. These findings suggest that low-dose dexmedetomidine infusion during laparoscopy-assisted gastrectomy exerts a minimal anti-inflammatory effect (based on the alleviation of IL-6 elevation at the end of surgery); this minimal effect can be certified by the non-significant and minimal increase in the CRP level noted postoperatively in this study. Further prospective, double-blinded, randomized clinical trials are required to gain a clearer understanding of the anti-inflammatory effects of dexmedetomidine.

## Figures and Tables

**Table 1:** Patient and surgical characteristics.

Variable	Control Group (*n* = 42)	Dexmedetomidine Group (*n* = 42)	*p*-Value
Age (yr)	59.4 ± 10.0	57.3 ± 9.2	0.322
Male sex	24 (57%)	19 (45%)	0.275
Body mass index (kg/m^2^)	23.7 ± 2.5	23.4 ± 2.8	0.677
ASA physical status			0.329
I	4 (10%)	8 (19%)	
II	27 (64%)	27 (64%)	
III	11 (26%)	7 (17%)	
Co-morbidities			
Hypertension	23 (55%)	28 (67%)	0.264
Diabetes mellitus	34 (81%)	38 (90%)	0.212
Type of operation			0.367
Subtotal gastrectomy	34 (81%)	58 (87%)	
Total gastrectomy	6 (14%)	2 (5%)	
Proximal subtotal gastrectomy	2 (5%)	3 (7%)	
Type of reconstruction and anastomosis			0.918
Billoth I	23 (55%)	23 (55%)	
Billoth II	6 (14%)	8 (19%)	
Double tract	2 (5%)	2 (5%)	
Roux-en-Y	11 (26%)	9 (21%)	
Extent of lymph node dissection			0.434
D1	31 (74%)	34 (81%)	
D2	11 (26%)	8 (19%)	
TNM stage			0.248
I	34 (81%)	38 (90%)	
II	4 (10%)	4 (10%)	
III	3 (7%)	0 (0%)	
IV	1 (2%)	0 (0%)	

Values are presented as mean ± standard deviation or as number of patients (percentage). ASA: American Society of Anesthesiologists; TNM: tumor node metastasis.

**Table 2:** Intraoperative variables.

Variable	Control Group (*n* = 42)	Dexmedetomidine Group (*n* = 42)	*p*-Value
Anesthesia time (min)	222 ± 56	203 ± 48	0.106
Operation time (min)	188 ± 57	173 ± 46	0.176
Pneumoperitoneum time (min)	156 ± 55	143 ± 46	0.211
Fluid intake (mL)	1554 ± 496	1485 ± 385	0.478
Blood loss (mL)	91 ± 127	60 ± 44	0.138
Urine output (mL)	230 ± 215	179 ± 73	0.150
Administered dose of remifentanil (μg)	800 ± 337	543 ± 168	<0.001 *
Administered dose of ephedrine (mg)	6.4 ± 5.5	12.5 ± 6.9	<0.001 *
Administered dose of phenylephrine (mg)	0.7 ± 1.2	0.6 ± 1.1	0.667

Values are presented as mean ± standard deviation or as number of patients (percentage). * *p* < 0.001.

**Table 3:** Postoperative variables.

Variable	Control Group (*n* = 42)	Dexmedetomidine Group (*n* = 42)	*p*-Value
Length of hospital stay (days)	6.6 ± 4.0	5.7 ± 1.9	0.174
Final pathology			0.803
Tubular adenocarcinoma			
Well differentiated	4 (10%)	6 (14%)	
Moderately differentiated	5 (12%)	7 (17%)	
Poor differentiated	18 (43%)	16 (38%)	
Signet ring cell carcinoma	15 (36%)	13 (31%)	
Surgical complications			0.424
Intraabdominal abscess	1 (2%)	0 (0%)	
Anastomosis leakage	2 (5%)	1 (2%)	
Wound infection	1 (2%)	0 (0%)	
None	38 (90%)	41 (98%)	
PCA-related complications			0.249
Nausea and/or vomiting	6 (14%)	12 (29%)	
Dizziness	10 (24%)	10 (24%)	
None	26 (62%)	20 (48%)	

Values are presented as mean ± standard deviation or as number of patients (percentage). PCA: patient-controlled analgesia.

**Table 4:** Inflammatory indices.

Variable	Control Group (*n* = 42)	Dexmedetomidine Group (*n* = 42)	*p*-Value (Group × Time)
IL-6 (ng/mL)			0.019
Pre-OP	1.9 ± 2.2	1.8 ± 1.5	
Pneumo 90 min	5.9 ± 5.7	4.7 ± 3.3	
OP end	36.2 ± 32.1	22.0 ± 12.5	
CRP (mg/L)			0.106
Pre-OP	1.5 ± 0.5	1.1 ± 0.3	
POD 0	2.5 ± 0.6	1.3 ± 0.2	
POD 1	38.6 ± 3.8	30.2 ± 2.6	
POD 2	89.9 ± 8.5	74.8 ± 7.5	
POD 3	94.8 ± 11.5	73.2 ± 8.2	
POD 5	85.6 ± 14.8	54.7 ± 8.0	
POD 30	14.0 ± 5.9	6.8 ± 2.8	
POD 90	4.0 ± 2.0	1.6 ± 0.5	
WBC count (/µL)			0.076
Pre-OP	6.3 ± 0.3	6.4 ± 0.3	
POD 0	12.6 ± 0.5	11.3 ± 0.5	
POD 1	10.4 ± 0.5	9.6 ± 0.4	
POD 2	9.0 ± 0.6	7.7 ± 0.4	
POD 3	7.6 ± 0.5	6.4 ± 0.3	
POD 5	6.8 ± 0.4	6.2 ± 0.3	
POD 30	7.2 ± 0.7	5.9 ± 0.3	
POD 90	5.7 ± 0.3	5.6 ± 0.2	

Values are presented as mean ± standard deviation or as number of patients (percentage). Pre-OP, pre-operation; Pneumo 90 min, 90 min after the start of pneumoperitoneum; OP end, at the end of the surgery; POD, postoperative day; IL-6, interleukin-6; CRP, C-reactive protein; WBC, white blood cell.

## Data Availability

The datasets used and/or analyzed during the current study are available from the corresponding author on reasonable request.

## References

[1] Takahashi Y., Kawamura M., Hato T., Harada M., Matsutani N., Horio H. (2016). Neutrophil-lymphocyte ratio as a prognostic marker for lung adenocarcinoma after complete resection. World J. Surg..

[2] Zhang W.W., Liu K.J., Hu G.L., Liang W.J. (2015). Preoperative platelet/lymphocyte ratio is a superior prognostic factor compared to other systemic inflammatory response markers in ovarian cancer patients. Tumour Biol. J. Int. Soc. Oncodev. Biol. Med..

[3] Xiao W.K., Chen D., Li S.Q., Fu S.J., Peng B.G., Liang L.J. (2014). Prognostic significance of neutrophil-lymphocyte ratio in hepatocellular carcinoma: A meta-analysis. BMC Cancer.

[4] Longhini F., Bruni A., Garofalo E., De Sarro R., Memeo R., Navalesi P., Navarra G., Ranieri G., Currò G., Ammendola M. (2020). Anesthetic strategies in oncological surgery: Not only a simple sleep, but also impact on immunosuppression and cancer recurrence. Cancer Manag. Res..

[5] Anand S., Bhatia A., Rajkumar, Sapra H., Gupta V., Mehta Y. (2012). Dexmedetomidine for monitored anesthesia care in patients undergoing liberation procedure for multiple sclerosis: An observational study. Saudi J. Anaesth..

[6] Horowitz M., Neeman E., Sharon E., Ben-Eliyahu S. (2015). Exploiting the critical perioperative period to improve long-term cancer outcomes. Nat. Rev. Clin. Oncol..

[7] Bhana N., Goa K.L., McClellan K.J. (2000). Dexmedetomidine. Drugs.

[8] Li Y., He R., Chen S., Qu Y. (2015). Effect of dexmedetomidine on early postoperative cognitive dysfunction and peri-operative inflammation in elderly patients undergoing laparoscopic cholecystectomy. Exp. Ther. Med..

[9] Taniguchi T., Kidani Y., Kanakura H., Takemoto Y., Yamamoto K. (2004). Effects of dexmedetomidine on mortality rate and inflammatory responses to endotoxin-induced shock in rats. Crit. Care Med..

[10] Xu L., Bao H., Si Y., Wang X. (2013). Effects of dexmedetomidine on early and late cytokines during polymicrobial sepsis in mice. Inflamm. Res..

[11] Dong W., Chen M.H., Yang Y.H., Zhang X., Huang M.J., Yang X.J., Wang H.Z. (2017). The effect of dexmedetomidine on expressions of inflammatory factors in patients with radical resection of gastric cancer. Eur. Rev. Med. Pharmacol. Sci..

[12] Gabriel A., Gupta S., Orgill D.P. (2019). Challenges and management of surgical site occurrences. Plast. Reconstr. Surg..

[13] Ni Choileain N., Redmond H.P. (2006). Cell response to surgery. Arch. Surg..

[14] Gabay C., Kushner I. (1999). Acute-phase proteins and other systemic responses to inflammation. N. Engl. J. Med..

[15] Povoa P. (2002). C-reactive protein: A valuable marker of sepsis. Intensive Care Med..

[16] Goldfarb Y., Sorski L., Benish M., Levi B., Melamed R., Ben-Eliyahu S. (2011). Improving postoperative immune status and resistance to cancer metastasis: A combined perioperative approach of immunostimulation and prevention of excessive surgical stress responses. Ann. Surg..

[17] Kurosawa S., Kato M. (2008). Anesthetics, immune cells, and immune responses. J. Anesth..

[18] Lee J.W., Shahzad M.M., Lin Y.G., Armaiz-Pena G., Mangala L.S., Han H.D., Kim H.S., Nam E.J., Jennings N.B., Halder J. (2009). Surgical stress promotes tumor growth in ovarian carcinoma. Clin. Cancer Res. Off. J. Am. Assoc. Cancer Res..

[19] Rosenberger P.H., Ickovics J.R., Epel E., Nadler E., Jokl P., Fulkerson J.P., Tillie J.M., Dhabhar F.S. (2009). Surgical stress-induced immune cell redistribution profiles predict short-term and long-term postsurgical recovery. A prospective study. J. Bone Jt. Surg. Am. Vol..

[20] Veenhof A.A., Sietses C., von Blomberg B.M., van Hoogstraten I.M., vd Pas M.H., Meijerink W.J., vd Peet D.L., vd Tol M.P., Bonjer H.J., Cuesta M.A. (2011). The surgical stress response and postoperative immune function after laparoscopic or conventional total mesorectal excision in rectal cancer: A randomized trial. Int. J. Color. Dis..

[21] Wang K., Wu M., Xu J., Wu C., Zhang B., Wang G., Ma D. (2019). Effects of dexmedetomidine on perioperative stress, inflammation, and immune function: Systematic review and meta-analysis. Br. J. Anaesth..

[22] Wang K., Li C. (2018). Effects of dexmedetomidine on inflammatory factors, t lymphocyte subsets and expression of nf-kappab in peripheral blood mononuclear cells in patients receiving radical surgery of colon carcinoma. Oncol. Lett..

[23] Wang Y., Xu X., Liu H., Ji F. (2015). Effects of dexmedetomidine on patients undergoing radical gastrectomy. J. Surg. Res..

[24] Tanabe K., Matsushima-Nishiwaki R., Kozawa O., Iida H. (2014). Dexmedetomidine suppresses interleukin-1beta-induced interleukin-6 synthesis in rat glial cells. Int. J. Mol. Med..

[25] Zhou X.Y., Liu J., Xu Z.P., Fu Q., Wang P.Q., Zhang H. (2019). Dexmedetomidine inhibits the lipopolysaccharide-stimulated inflammatory response in microglia through the pathway involving tlr4 and nf-κb. Kaohsiung J. Med. Sci..

[26] Dutta S., Lal R., Karol M.D., Cohen T., Ebert T. (2000). Influence of cardiac output on dexmedetomidine pharmacokinetics. J. Pharm. Sci..

[27] Jorm C.M., Stamford J.A. (1993). Actions of the hypnotic anaesthetic, dexmedetomidine, on noradrenaline release and cell firing in rat locus coeruleus slices. Br. J. Anaesth..

[28] Lee J., Hwang H.W., Jeong J.Y., Kim Y.M., Park C., Kim J.Y. (2022). The effect of low-dose dexmedetomidine on pain and inflammation in patients undergoing laparoscopic hysterectomy. J. Clin. Med..

[29] Bekker A., Haile M., Kline R., Didehvar S., Babu R., Martiniuk F., Urban M. (2013). The effect of intraoperative infusion of dexmedetomidine on the quality of recovery after major spinal surgery. J. Neurosurg. Anesth..

[30] Moon J., Yoo Y.C., Kim M.H., Jeon S., Joo H.J., Chun D.H., Kim N.Y. (2021). Administration of low-dose dexmedetomidine did not affect acute inflammatory response after cytoreductive surgery combined with hyperthermic intraperitoneal chemotherapy: A double-blind randomized controlled trial. J. Clin. Med..

[31] Yu H., Dong R., Lu Y., Yang X., Chen C., Zhang Z., Peng M. (2017). Short-term postoperative cognitive dysfunction and inflammatory response in patients undergoing cytoreductive surgery and hyperthermic intraperitoneal chemotherapy: A pilot study. Mediat. Inflamm..

[32] Bulow N.M., Colpo E., Pereira R.P., Correa E.F., Waczuk E.P., Duarte M.F., Rocha J.B. (2016). Dexmedetomidine decreases the inflammatory response to myocardial surgery under mini-cardiopulmonary bypass. Braz. J. Med. Biol. Res..

[33] Ueki M., Kawasaki T., Habe K., Hamada K., Kawasaki C., Sata T. (2014). The effects of dexmedetomidine on inflammatory mediators after cardiopulmonary bypass. Anaesthesia.

[34] Sietses C., Wiezer M.J., Eijsbouts Q.A., Beelen R.H., van Leeuwen P.A., von Blomberg B.M., Meijer S., Cuesta M.A. (1999). A prospective randomized study of the systemic immune response after laparoscopic and conventional nissen fundoplication. Surgery.

[35] Okholm C., Goetze J.P., Svendsen L.B., Achiam M.P. (2014). Inflammatory response in laparoscopic vs. Open surgery for gastric cancer. Scand. J. Gastroenterol..

[36] Sista F., Schietroma M., Santis G.D., Mattei A., Cecilia E.M., Piccione F., Leardi S., Carlei F., Amicucci G. (2013). Systemic inflammation and immune response after laparotomy vs laparoscopy in patients with acute cholecystitis, complicated by peritonitis. World J. Gastrointest. Surg..

[37] Targarona E.M., Pons M.J., Balagué C., Espert J.J., Moral A., Martínez J., Gaya J., Filella X., Rivera F., Ballesta A. (1996). Acute phase is the only significantly reduced component of the injury response after laparoscopic cholecystectomy. World J. Surg..

[38] Harmon G.D., Senagore A.J., Kilbride M.J., Warzynski M.J. (1994). Interleukin-6 response to laparoscopic and open colectomy. Dis. Colon Rectum.

[39] Xie Y., Jiang W., Zhao L., Wu Y., Xie H. (2020). Effect of dexmedetomidine on perioperative inflammation and lung protection in elderly patients undergoing radical resection of lung cancer. Int. J. Clin. Exp. Pathol..

[40] Gurbet A., Basagan-Mogol E., Turker G., Ugun F., Kaya F.N., Ozcan B. (2006). Intraoperative infusion of dexmedetomidine reduces perioperative analgesic requirements. Can. J. Anaesth. J. Can. D’anesthesie.

[41] Mahmoud M., Sadhasivam S., Salisbury S., Nick T.G., Schnell B., Sestokas A.K., Wiggins C., Samuels P., Kabalin T., McAuliffe J. (2010). Susceptibility of transcranial electric motor-evoked potentials to varying targeted blood levels of dexmedetomidine during spine surgery. Anesthesiology.

[42] Ngwenyama N.E., Anderson J., Hoernschemeyer D.G., Tobias J.D. (2008). Effects of dexmedetomidine on propofol and remifentanil infusion rates during total intravenous anesthesia for spine surgery in adolescents. Paediatr. Anaesth..

[43] Le Guen M., Liu N., Tounou F., Augé M., Tuil O., Chazot T., Dardelle D., Laloë P.A., Bonnet F., Sessler D.I. (2014). Dexmedetomidine reduces propofol and remifentanil requirements during bispectral index-guided closed-loop anesthesia: A double-blind, placebo-controlled trial. Anesth. Analg..

[44] Blaudszun G., Lysakowski C., Elia N., Tramèr M.R. (2012). Effect of perioperative systemic α2 agonists on postoperative morphine consumption and pain intensity: Systematic review and meta-analysis of randomized controlled trials. Anesthesiology.

[45] Cheung C.W., Qiu Q., Ying A.C., Choi S.W., Law W.L., Irwin M.G. (2014). The effects of intra-operative dexmedetomidine on postoperative pain, side-effects and recovery in colorectal surgery. Anaesthesia.

[46] Ren C., Chi M., Zhang Y., Zhang Z., Qi F., Liu Z. (2015). Dexmedetomidine in postoperative analgesia in patients undergoing hysterectomy: A consort-prospective, randomized, controlled trial. Medicine.

[47] Arain S.R., Ruehlow R.M., Uhrich T.D., Ebert T.J. (2004). The efficacy of dexmedetomidine versus morphine for postoperative analgesia after major inpatient surgery. Anesth. Analg..

[48] Kalaskar V.P., Ruparel D.H., Wakode R.P. (2021). Effects of dexmedetomidine infusion in low dose on dose reduction of propofol, intraoperative hemodynamics, and postoperative analgesia in patients undergoing laparoscopic cholecystectomy. Anesth. Essays Res..

